# Hypoglycemic Effect of a Combined *Andrographis paniculata* and *Caesalpinia sappan* Extract in Streptozocin-Induced Diabetic Rats

**DOI:** 10.1155/2020/8856129

**Published:** 2020-11-10

**Authors:** Febrika Wediasari, Gumilar A. Nugroho, Zahra Fadhilah, Berna Elya, Heri Setiawan, Tjandrawati Mozef

**Affiliations:** ^1^Laboratory of Phytochemistry, Faculty of Pharmacy, Universitas Indonesia, Depok 16424, Indonesia; ^2^Laboratory of Pharmacology, Faculty of Pharmacy, Universitas Indonesia, Depok 16424, Indonesia; ^3^Indonesian Institute of Sciences, Jakarta, Indonesia

## Abstract

**Introduction:**

Researchers usually use herbal combinations to explore and develop traditional medicine to obtain additional benefits in the treatment of diseases, including diabetes. This study aims to evaluate the hypoglycemic effect of the combination of *Andrographis paniculata* (Burm. f.) Wall ex Nees and *Caesalpinia sappan* Linn extract (APCSE) on diabetes-induced rats. There has not been sufficient research on this combination; however, single extract studies of these plants have been widely conducted.

**Materials and Methods:**

Male Sprague Dawley rats (160–200 g) were induced by injecting a low dose of streptozotocin (35 mg/kg BW) twice and fed with a high-fat diet containing 25% fat, whereas control animals received only standard feed. Rats were treated with APCSE at doses of 100 mg and 200 mg/kg BW for seven days and compared to the APE and CSE groups treated with the extract at 100 mg, respectively. For the control group, rats were treated with metformin with a dose of 250 mg/kg. The antihyperglycemic and antihyperlipidemic effects were determined by measuring blood glucose levels and lipid profiles (cholesterol, triglycerides, HDL, and LDL). To assess the impact of the extract on pancreatic and adipose tissue, the number of pancreatic beta cells and adipocytes was evaluated through histopathological and immunohistochemical study. *Results and Discussion*. In a nonfasting state, the blood glucose change in APCSE 200 mg was 18.65% and was significantly lower from the DM group. However, a single extract of APE and CSE showed lower fasting blood glucose levels compared to the combined extract. Lipid profiles show no significant differences in cholesterol levels between groups; however, all treatment groups, including metformin, showed higher triglyceride levels. The APE-treated group showed significantly lower HDL and LDL, whereas CSE only showed lower LDL. The *β*-cell number was significantly higher after treatment with single extract CSE. The CSE and the combined extract groups showed hyperplasia adipocytes.

**Conclusion:**

The combined extract of APCSE has a moderate antihyperglycemic effect; however, a single extract may have better potential than the combined extract.

## 1. Introduction

Diabetes mellitus (DM) is a collection of disorders characterized by hyperglycemia and glucose intolerance and caused by insulin deficiency, insulin resistance, or both [1]. A tremendous number of people in the world nowadays live with DM, which makes it one of the global health threats recognized by the WHO. DM causes death and disability and decreases life expectancy. Based on data from the International Diabetes Federation in 2017, Indonesia has the sixth largest population of people with diabetes globally, following China, India, the United States, Brazil, and Mexico. In total, 10.3 million were estimated to have diabetes in Indonesia in 2017, and this figure is projected to increase to 16.7 million by the year 2045 [2].

Plants have been used as sources of traditional medicine for thousands of years, and research had identified plants as good sources of new remedies for many life-threatening diseases. Plants with antidiabetic properties are believed to be useful sources for the development of effective oral hypoglycemic agents. Indonesia is rich in biodiversity, and several plants such as *Andrographis paniculata* (Burm. f.) Wall ex Nees and *Caesalpinia sappan* Linn have been demonstrated as potential sources of antidiabetic properties [3, 4].

*Andrographis paniculata* (AP) originated from India is widely grown in Southeast Asian countries including Indonesia, and it is often used in jamu pahitan (Indonesian traditional medicine) to maintain health and treat many health problems such as tonsillitis, chancre, typhoid fever, diabetes, eczema, the common cold, diphtheria, depurative, epilepsy, gonorrhea, syphilis, and dandruff [3]. The extract of AP (APE) demonstrated to reduce blood glucose levels in diabetic rats in several studies [5, 6]. An *in vitro* study of the extract revealed a strong insulin-secreting effect in BRIN-BD11 cells, a pancreatic cell line expressing insulin, and glucokinase [7]. The main active compound in APE is andrographolide, and it excreted a hypoglycemic effect by increasing the mRNA and protein levels of GLUT4 in rats with type 1 DM [8].

*Caesalpinia sappan* (CS) is a plant from the leguminous family that is commonly known as brazilwood or sappanwood and distributed across Southeast Asia. The dried heartwood of CS is considered a depurative and is used to treat hemoptysis, syphilis, conjunctivitis, dysentery, diarrhea, dysentery, and tuberculosis [4]. CS contains the flavonoid brazilin, and a previous study found that the compound increases glucose transport by recruiting GLUT4. Research reports that brazilin lowers blood glucose levels through an extrapancreatic action. Brazilin increases the peripheral utilization and activities of glucose metabolic enzymes. You et al. [9] discovered that brazilin (1–100 *μ*M) concentration-dependently increases the production of fructose-2,6-bisphosphate (F-2,6-BP) in hepatocytes by elevating the intracellular levels of fructose-6-phosphate and hexose-6-phosphate. Brazilin also significantly increases 6-phospho-2-kinase (PEK-2) and pyruvate kinase activity. A recent study illustrated that the extract of CS (CSE) works by inhibiting dipeptidyl-peptidase IV [10].

Research on this combination effect has never been carried out before; from previous studies, it was known that the mechanism of action of each differs. By combining the two extracts at the same amount, we expect to evaluate the hypoglycemic effect of their combination in diabetic rats. Antihyperglycemic and antihyperlipidemic results were determined by measuring blood glucose levels and lipid profiles (cholesterol, triglyceride, HDL, and LDL). The number of pancreatic beta cells and adipocyte was further evaluated to understand the effect of the extract in the pancreas and adipose tissue through histopathology and immunohistochemistry.

## 2. Materials and Methods

### 2.1. Plant Material and Extract Preparation

Plants were collected from Wonogiri, Central Java, Indonesia, and identified by the Indonesian Institute of Sciences, Research Center for Plant Conservation, and Botanic Gardens, Bogor, West Java (B.2405/IPH.3/KS/VII/2019). The aerial parts of AP and the heartwood of CS were dried and powdered. Each extract was prepared separately. APE was prepared by extracting 1.5 kg of powdered AP material with 5.0 L of 70% ethanol. The remaining powdered residues were reextracted with 5.0 L of ethanol, combined with the first extract, concentrated, and dried using a rotary evaporator at <50°C. CSE was prepared by extracting 2.5 kg of powdered CS material with 5.0 L of 70% ethanol. The remaining powdered residues were reextracted with 5.0 L of ethanol, combined with the first extract, concentrated, and dried using a rotary evaporator at <50°C. The % yield of APE was 16.21%, and CSE was 12.71% per dried weight. Each extract was analyzed for the phytochemical existence of alkaloids, tannins, saponins, flavonoids, phenolic compounds, triterpenoid, steroids, and glycoside.

### 2.2. Animals

Sprague Dawley (SD) male rats (6–8 weeks old, 150–200 g) were obtained from the National Agency of Drug and Food Control. Rats were acclimatized with free access to food and water for at least ten days and maintained under constant temperature (22 ± 2°C), humidity (70 ± 10%), and light-dark cycle (12 h/12 h) at the Tropical Biopharmaca Research Center. This study was approved by the Ethics Committee of the Faculty of Medicine University of Indonesia (KET-894/UN2.F1/ETIK/PPM.00.02/2019). Animal handling was performed according to the procedures outlined by the National Agency of Drug and Food Control Republic of Indonesia No.7 for the year 2014. Five rats were randomly selected to be in the normal control (NC) group and received a normal diet (ND); forty other rats received high-fat diet (HFD) with feed containing 25% fat.

### 2.3. Acute Oral Toxicity Study

The acute toxicity study was conducted according to the Organization for Economic Co-operation and Development (OECD) guideline 420 (fixed-dose procedure). The acute oral toxicity is studied by stepwise procedure using fixed doses of 300 mg and 2000 mg/kg BW of the combined extract. The initial dose level was selected based on a sighting study of 300 mg/kg BW, and the maximum dose was 2000 mg/kg BW. Animals may be dosed at higher or lower fixed dose depending on the presence or absence of signs of toxicity or mortality. It was observed that the test combined extract was not mortal, even at 2000 mg/kg BW dose.

### 2.4. Induction of Diabetes

Diabetes was induced via intraperitoneal injections of streptozotocin/STZ *N*-(methyl nitrosocarbamoyl)-*α*-D-glucosamine (Wako, Fujifilm, Japan). Rats were fasted overnight before STZ administration. The next day, rats were injected with 35 mg/kg BW in 0.1 M cold sodium citrate. Diabetes was identified by the presence of polydipsia, polyuria, and fasting blood glucose level of ≥150 mg/mL [11, 12].

#### 2.4.1. Procedure for Antidiabetic Activity

Forty-five rats were divided into seven groups; each group had 5 SD rats, group 1 consisted of the normal control group (*n* = 5). Group 2 consisted of diabetic rats (rats that have been treated with multiple doses of STZ). Group 3 consisted of diabetic rats that received metformin (250 mg/kg BW) as positive control. Group 4 diabetic rats received 100 mg of APE. Group 5 diabetic rats received 100 mg of CSE. Group 6 diabetic rats received 100 mg of APCSE. Group 7 diabetic rats received 200 mg of APCSE.

### 2.5. Measurement of Blood Glucose and Body Weight

Blood samples were drawn from each group to measure blood glucose. Blood glucose was measured with and without fasting; fasting blood glucose was taken after rats were fasted overnight. Blood drop was taken from the distal end of the tail, applied to a test strip, and analyzed immediately using a blood glucose monitoring device (Accu-Chek Active, Roche Diagnostics, Mannheim, Germany). By the end of the study, rats were euthanized under ketamine and xylazine anesthesia. Blood samples were collected via cardiac puncture into tubes, centrifuged, and evaluated for lipid profile: total cholesterol, triglyceride, high-density lipoprotein (HDL), and low-density lipoprotein (LDL).

### 2.6. Histological and Immunohistochemistry Observation

The adipose and the pancreatic tissue were excised, rinsed in ice-cold saline, and stored in formalin solution for further histopathological studies. The pancreatic tissue was fixed with 4% paraformaldehyde in phosphate-buffered saline for at least two hours. The tissue was prepared as a section slide for immunohistochemistry (IHC) analysis using primary anti-insulin (clone K36AC10). Hematoxylin-eosin staining was used to analyze and evaluated under the light microscope (Olympus B1, Japan) [13].

### 2.7. Statistical Analysis

Data were analyzed using GraphPad Prism 8^th^ edition and expressed as the mean ± SEM. Normality was tested using the Shapiro–Wilk test. Data were analyzed using ANOVA, followed by the least significant difference (LSD) or Kruskal–Wallis test followed by Dunn's test. The LSD or Dunn test was used to assess statistical significance compared with NC or DM groups. The difference was considered statistically significant at *p* < 0.05.

## 3. Results

### 3.1. Phytochemical Analysis

Identification of bioactive compounds revealed that both extracts contained phenolic compounds, triterpenoids, glycosides, and steroids. However, only the AP contains alkaloids, saponins, and tannins.

### 3.2. Body Weight and Blood Glucose

**Figure 1:**
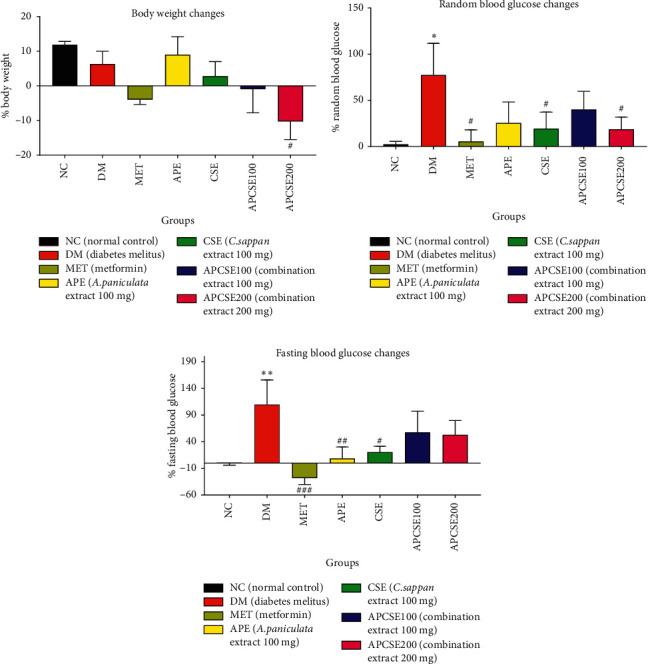
(a) Body weight changes, (b) random (nonfasting) blood glucose, and (c) fasting blood glucose. Changes in rats. The results are presented as the mean ± SEM (*n* = 5). Significantly different from NC (^*∗*^*p* < 0.05;^∗∗^*p* < 0.01) and significantly different from DM (^#^*p* < 0.05;^##^*p* < 0.01).

Changes in body weight, random blood glucose (RBG), and fasting blood glucose (FBG) levels were measured in rats following treatment with APCSE (Figure 1). Changes are expressed in percentage of different values before and after treatment, then it was compared with the value before treatment. Positive changes showed an increase in measured variables. Conversely, negative changes showed a decrease. Rat body weight increased in the NC, DM, APE, and CSE groups, whereas treatment with MET and both APCSE 100 and 200 mg showed a decrease in body weight. However, only APCSE 200 mg group (−10.28 ± 5.24%, *p* < 0.05) was significantly different from DM and control.

RBG levels were measured in nonfasting conditions. Significant differences were observed between the NC and DM groups (77.41 ± 34.32%, *p* < 0.05). All treatment groups showed an increase in RBG; however, the increments were not as high as the DM group. Changes in blood glucose levels in the MET (5.61 ± 12.44%, *p* < 0.05), CSE (19.37 ± 17.86%, *p* < 0.05), and APCSE200 (18.65 ± 13.16%, *p* < 0.05) groups were significantly lower compared to the DM group.

FBG levels were measured after an overnight fast (16 h). DM group showed significantly higher changes in FBG (109.46 ± 46.39%, *p* < 0.05) compared to the NC group. Only the MET group showed negative changes, and therefore, it differed significantly from the DM group (−28.16 ± 12.26%, *p* < 0.01). All animal groups treated with the extract showed positive changes of FBG; however, APE (8.47 ± 21.57%, *p* < 0.01) and CSE (20.50 ± 10.88%, *p* < 0.05) showed only a little increase and differed significantly from the DM group. APCSE groups showed a rise in FBG, not as high as the DM group, but could not be distinguished statistically.

### 3.3. Lipid Profiles

Rats were sacrificed at the end of treatment; blood samples were collected through cardiac puncture. Cholesterol, triglyceride, HDL, and LDL were then measured to depict the metabolic profile of lipids.  Table 1 shows the result of the measurement, and these are expressed in mean ± SEM (mg/dL).

Cholesterol levels did not significantly differ among the groups during the observation period. However, triglyceride levels were significantly higher in the CSE (188.70 ± 42.00, *p* < 0.05) and APCSE100 groups (176.58 ± 35.42, *p* < 0.01) than in the normal group. HDL levels were significantly lower in the APE group (55.92 ± 3.58, *p* < 0.05) than in the NC group. LDL levels were significantly lower in the CSE group (37.99 ± 2.43, *p* < 0.05) than in the NC group. Conversely, LDL levels in the APCSE100 and APCSE200 groups were similar to those in the MET group.

### 3.4. Histopathological Examination

**Figure 2:**
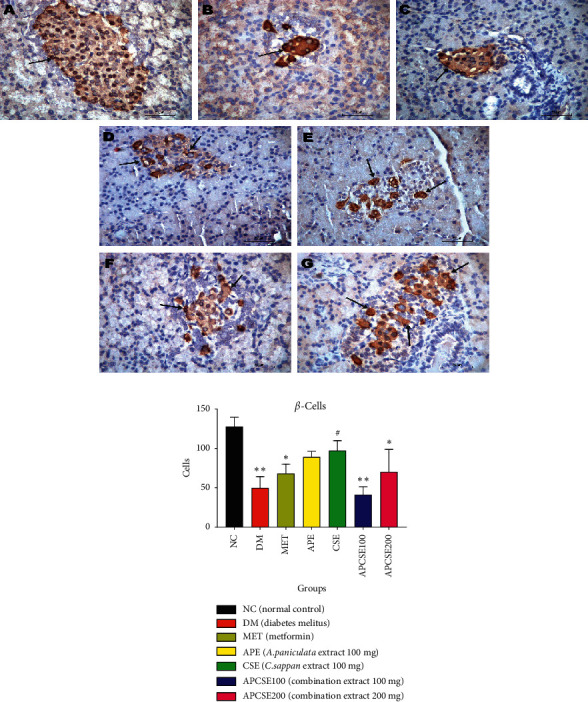
(a) Immunohistochemistry staining of pancreatic islets. (A) Section of the pancreas of the NC group showing immunoreactivity of insulin in *β*-cells occupying most of the islets; (B) the pancreas of the DM group showing a reduction in the immunohistochemistry expression of insulin in *β*-cells; (C) section from the MET group (diabetic rats + metformin) showing an apparent increase in the number and area of *β*-cells compared with the DM group; (D) section from the APE group (diabetic rats + APE 100 mg) showing an apparent increase in the number and area of *β*-cells compared with the DM group; (E) pancreas from the CSE group (diabetic rats + CSE 100 mg) showing an apparent increase in the number and area of *β*-cells compared with DM group; (F) section from the APCSE100 group (diabetic rats + APCSE 100 mg) showing a lower density of insulin expressing *β*-cells; (G) the area from the APCSE200 group (diabetic rats + APCSE 200 mg) showing an increase in insulin expressing *β*-cells with normal density. (b) *β*-Cell count of the pancreas. Results presented are mean ± SEM; significantly different from DM (^#^*p* < 0.05;^##^*p* < 0.01) and significantly different from normal (^*∗*^*p* < 0.05;^∗∗^*p* < 0.01).

The histopathological examination was performed after the last day of treatment. The immunohistochemistry staining of pancreatic tissue is presented in Figure 2(a), and the *β*-cell count is presented in Figure 2(b). The immunochemistry staining in Figure 2(a) clearly shows that there was a notable reduction in the pancreatic *β*-cells from DM rats compared to normal rats; the *β*-cell count from both groups differed significantly. The rats from the APE (89.13 ± 7.13) and CSE (97.17 ± 12.62, *p* < 0.05) groups showed a higher *β*-cell count than the rats from the DM group; the CSE group showed a significant difference. Meanwhile, the pancreatic *β*-cell count from the APCSE100 (41.30 ± 10.06) and APSCE200 (70.23 ± 28.48) groups showed a similar number to the DM group (49.87 ± 14.34).

**Figure 3:**
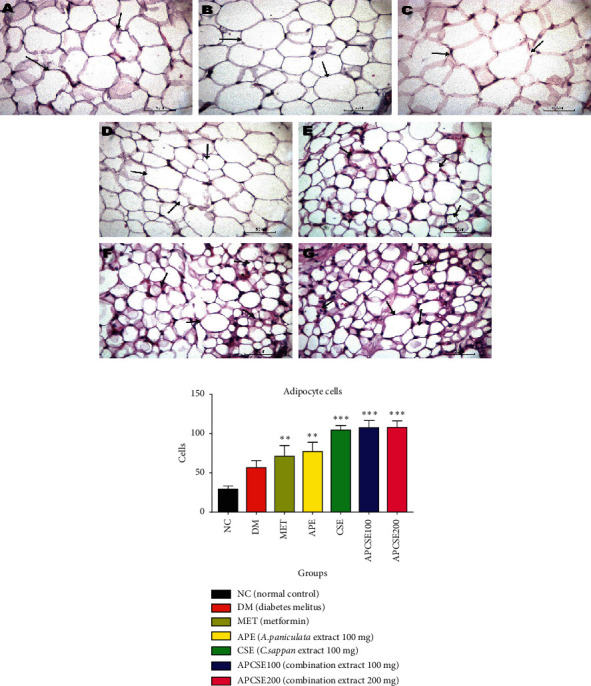
(a) Histological interpretation of the adipose tissue in rats (H&E, 40x). (A) Section from the NC group showing large adipocytes of normal rats; (B) section from diabetic rats of the DM group showing large adipocytes cells; (C) section from the MET group (diabetic rats + metformin) showing large adipocytes; (D) section from APE groups (diabetic rats + APE 100 mg) showing mixed adipocytes large and small, mostly massive; (E) CSE group (diabetic rats + CSE 100 mg) showing mixed adipocytes with large and small cells, mostly small sizes; (F) APCSE100 and (G) APCSE200 offer diverse cell sizes, but most are the smaller size of adipocytes. (b) Adipocyte cell count. Adipocyte cell count in the CSE, APCSE100, and APCSE200 groups was significantly different compared to the DM group. Results presented are mean ± SEM; significantly different from DM (^#^*p* < 0.05;^##^*p* < 0.01) and significantly different from normal (^*∗*^*p* < 0.05;^∗∗^*p* < 0.01).

Figure 3(a) shows the histopathology of the adipocyte from the adipose tissue. Compared to the NC group, all groups with diabetic induction resulted in a higher number of adipocyte. Adipocyte counts were significantly higher in the CSE (104.90 ± 5.30, *p* < 0.01), APCSE 100 (107.90 ± 8.71, *p* < 0.01), and APCSE 200 groups (108.20 ± 7.87, *p* < 0.01) compared to the normal and DM group. Interestingly, despite a higher number of adipocytes in the CSE, APCSE100, and APCSE200 groups, the size of adipocytes was significantly smaller than other groups.

## 4. Discussion

The use of APE or CSE alone demonstrated to improve diabetes in several studies. The research illustrated that APE works by inducing GLUT4 translocation, restoring islet cells, and upregulating antioxidant enzymes [14] and exerts cytoprotective effects [15], controlling glucose uptake and oxidation, restoring insulin signaling molecules in the liver, and decreasing the serum lipid profile [5]. Meanwhile, CSE inhibits *α*-glucosidase, and the extract has exhibited anti-inflammatory activity [16]. The heartwood extract inhibits the activity of DPP-IV [10]. Brazilin from CSE inhibits hepatic gluconeogenesis by elevating F-2,6-BP levels in hepatocytes, which may increase pyruvate kinase activity [9].

Each extract alone was reported safe; several studies said APE and CSE were safe up to 2000 mg/kg BW [17–20]. The combination of AP with other plants was reported safe and acted as promising antidiabetic effects. For example, AP and *Gynura procumbens* increased pancreatic insulin expression [13], whereas AP and *Centella asiatica* [7] exerted synergistic effects on blood cholesterol and HDL levels. The combination of AP and *Syzygium polyanthum* [21] improved the histopathology of islets. The APCSE combination in this present study shows the same it was safe up to 2000 mg/kg BB; the criteria reported practically nontoxic (LD50: 5–15 g).

The antidiabetic activity of the combination in this study was evaluated in rats fed with high-fat diet (25% fat) and induced twice with low doses of STZ to develop insulin resistance and dyslipidemia, creating type 2 diabetes rats [22, 23]. The HFD that we used was fed for three weeks, but the glucose level did not rise with HFD alone. By injecting the rats twice with dose injection of STZ, we established hyperglycemic rats and lipid profile higher than before the STZ injection.

STZ is a glucosamine-nitrosourea compound and alkylating agent that can damage the DNA of islet *β*-cells [24]. The diabetogenic properties of STZ are characterized by the selective destruction of *β*-cells, insulin deficiency, hyperglycemia, polydipsia, and polyuria and symptoms that resemble human diabetes. Most rats with STZ-induced diabetes exhibit decreased body weight. Although a low dose (e.g., 35 mg/dl) does not have significant effects on body weight [25], two injections at this dose could alter body weight. In the presence of insulin deficiency, proteins are degraded to provide amino acids for gluconeogenesis. This results in the loss of muscle mass and weight loss. This could explain the decline in weight in STZ-treated animals.

We discovered in this study the body weight of rats treated with the combined extract of APCSE 200 mg has reduced by 14.43%, significantly different compared to the DM group. The bodyweight of the APCSE 100 mg group and MET group had a decreasing number but not significant to DM. The single extract of APE and CSE tends to increase but not significant than the DM. Bodyweight increase of APE may be related to the adipocyte cells; the adipocyte cells were growing after the treatment, in contrast to APCSE, which has more increasing adipocyte cells but decreased body weight. The adipocytes in APCSE were smaller and had more vascularization.

Random blood glucose changes in this study discovered that all rats experience an increase in blood glucose. Blood glucose changes in MET (5.61 ± 34.32), CSE (19.37 ± 17.86), and APCSE 200 mg (18.65 ± 13.16) were significantly lower from DM with *p* < 0.05. The fasting blood glucose also experiences the same results, and the MET (−12.15 ± 12.26), APE (8.47 ± 21.57), and CSE (20.50 ± 10.88) were significantly different from the DM group, *p* < 0.05. Interestingly, the combined APCSE increased the fasting blood glucose. This finding was a contrast from other studies, but we realized our extract was not fractionated and may not produce the expected results. Rats were fasted overnight for about 16 hours, and fasting may have a role in the rising of blood glucose. When blood glucose is low, the body is compensating to produce glucagon hormone to produce glucose via gluconeogenesis. CSE acts as a DPP4 inhibitor [10], which can stimulate *β*-cells to produce glucagon and can be used to induce gluconeogenesis. On the other hand, the adipocyte in the CSE and APCSE groups was significantly higher compared to other groups. This condition may have a role in the rising of FBG.

Lipid profiles after treatment in this study presented in Table 1 show that there were no significant differences in cholesterol levels between groups; however, all treatment groups, including metformin, showed higher triglyceride levels. The triglyceride levels in APE (262.53 ± 159.34), CSE (188.7 ± 42.0), APCSE 100 mg (176.59 ± 35.42), and APCSE 200 mg (135.31 ± 27.76) were significantly different from the normal group (*p* < 0.05). The APE-treated group showed significantly lower HDL and LDL, whereas CSE only showed lower LDL.

The immunohistochemistry analysis shows that CSE has more *β*-cells than the DM group (97.17 ± 12.62, *p* < 0.05). The APE and APCSE also increased but not significant from the DM and NC groups; the *β*-cell count in the APCSE group was similar to the MET. Although there were more cell counts in CSE than in the APCSE, the insulin expression in APCSE shows more promising results.

The histopathology of adipocyte presented in Figure 3(a) shows the treated extract groups of APE, CSE, APCSE 100 mg, and 200 mg have more cells with smaller sizes; it seems that the adipocytes develop hyperplasia. In contrast to the control groups, the adipocyte size did not produce any smaller cells. Adipocyte cells contain lipid particle that serves as energy storage, and it also regulates glucose homeostasis [26]. Most PPAR-*γ* ligands are found mostly in the adipose tissue, like thiazolidinedione (TZD), and have excellent antidiabetic activity. The CSE and APCSE groups develop more adipocytes, much the same as troglitazone, which increases the number of small adipocytes without the change of white adipose tissue mass [27].

## 5. Conclusion

The present study using APCSE demonstrated the safety of the extract up to a dose of 2000 mg/kg BW. Blood glucose changes in APCSE (18.65 ± 13.16, *p* < 0.05) were significantly lower from DM in a nonfasting state. However, a single extract of APE and CSE showed lower fasting blood glucose compared to the combined extract. The *β*-cell number was significantly higher after treatment with single extract CSE. The immunohistochemistry *β*-cell count shows similar results to metformin. CSE and the combined extract groups showed adipocyte hyperplasia. As stated before, the combined extract of APCSE has a moderate antihyperglycemic effect, but the single extract may have better potential than the combined extract.

## Figures and Tables

**Table 1:** Lipid profiles after treatment.

Groups	Cholesterol (mg/dL)	Triglyceride (mg/dL)	HDL (mg/dL)	LDL (mg/dL)
NC (*n* = 5)	86.39 ± 4.59	83.85 ± 2.34	72.86 ± 4.77	55.05 ± 3.43
DM (*n* = 4)	88.05 ± 4.83	95.54 ± 7.56	72.89 ± 4.77	54.36 ± 3.27
MET (*n* = 5)	82.29 ± 10.46	117.71 ± 23.05	62.51 ± 8.86	46.25 ± 4.77
APE (*n* = 4)	74.70 ± 1.56	103.36 ± 9.41	55.92 ± 3.58^*∗*^	42.84 ± 1.29
CSE (*n* = 5)	89.33 ± 8.41	188.70 ± 42.00^∗∗#^	68.01 ± 4.45	37.99 ± 2.43^*∗*#^
APCSE100 (*n* = 4)	94.70 ± 5.05	176.58 ± 35.42^*∗*#^	70.49 ± 5.05	57.63 ± 17.52
APCSE200 (*n* = 5)	88.36 ± 4.84	107.93 ± 5.91	68.80 ± 3.62	49.82 ± 3.24

The results are presented as the mean ± SEM. Significantly different from NC (^*∗*^*p* < 0.05;^∗∗^*p* < 0.01) and significantly different from DM (^#^*p* < 0.05).

## Data Availability

The data that support the findings of this study are available from the corresponding author (berna.elya@farmasi.ui.ac.id) upon reasonable request.
